# Assessment of the Forces Developed During Orthodontic Treatment Using the Finite Element Method

**DOI:** 10.3390/dj14070406

**Published:** 2026-07-04

**Authors:** Maria Manuela Nardin, Mihaela-Roxana Brătoiu, Cristina Teodora Preoteasa, Diana-Elena Vlăduțu, Dragoș Laurențiu Popa, Alexandra Elena Done, Anne Marie Rauten, Felicia Ileana Mărășescu, Luminița Dăguci, Veronica Mercuț

**Affiliations:** 1 Department of Prosthodontics, University of Medicine and Pharmacy of Craiova, 200349 Craiova, Romania; nardin.mmanuela@gmail.com (M.M.N.); mihaelabotila09@yahoo.com (M.-R.B.); luminita.daguci@umfcv.ro (L.D.); veronica.mercut@umfcv.ro (V.M.); 2 Department of Scientific Research Methods-Ergonomics, Faculty of Dentistry, “Carol Davila” University of Medicine and Pharmacy, 020021 Bucharest, Romania; alexandra.done@drd.umfcd.ro; 3 Department of Automotive, Transportation and Industrial Engineering, Faculty of Mechanics, University of Craiova, 200478 Craiova, Romania; popadragoslaurentiu@yahoo.com; 4 Department of Orthodontics, University of Medicine and Pharmacy of Craiova, 200349 Craiova, Romania; annemarie.rauten@umfcv.ro (A.M.R.); ciuca_felicia@yahoo.com (F.I.M.)

**Keywords:** FEM, Ni-Ti archwire, orthodontic force, orthodontic treatment, bracket

## Abstract

**Background/Objectives:** This “in silico” study investigated the biomechanical behavior of fixed orthodontic systems by analyzing the forces generated by Ni–Ti archwires and their effects on the dento-maxillary system (DMS). The objectives were to estimate force levels for 0.012″, 0.014″, and 0.016″ archwires and to evaluate the resulting stress distribution, displacement and deformation, including the influence of dental malpositions such as infraposition and linguoposition on force transmission. **Methods:** A three-dimensional (3D) patient-specific model of the DMS was developed and finite element analysis (FEM) was performed. Orthodontic forces were determined analytically based on archwire deformation and applied as equivalent force systems at the bracket level. The analysis was performed under simplified assumptions, including linear elastic material behavior and the absence of bracket–archwire friction. **Results:** The results indicated a direct relationship between archwire diameter and force magnitude. Increased diameter was associated with higher stress, displacement and deformation values. Simulated infraposition and linguoposition produced localized variations in stress distribution, highlighting the influence of tooth position on biomechanical response. **Conclusions:** Within the limitations of the simplified analytical and numerical model, archwire diameter plays a significant role in determining force magnitude and stress distribution in orthodontic systems. The findings provide a comparative framework for understanding force transmission, although the results should be interpreted as theoretical estimates under idealized conditions rather than direct predictors of clinical behavior.

## 1. Introduction

Dento-maxillary anomalies exert negative psychological, social and physical effects, thereby diminishing oral health-related quality of life [1]. Within this context, the objectives of orthodontic therapy include the improvement in DMS function, the enhancement in psychosocial wellbeing and the mitigation of long-term risks associated with dento-maxillary anomalies. Such complications include dental wear, periodontal disease and pathological conditions due to the presence of impacted teeth [2].

Currently, the demand for orthodontic treatment has increased considerably, driven by the need to correct morphological discrepancies. This trend is further influenced by a range of factors, including sex, geographic origin, ethnicity [3] and socio-economic status [4].

Orthodontic treatment can be accomplished through a wide variety of removable, semi-removable and fixed appliances, sometimes used in conjunction with extraoral devices. Despite differences in their design and configuration, all these involve the controlled application of orthodontic forces acting upon the dentition and surrounding structures [5].

Dental movement represents a synergistic sequence of physical phenomena and biological tissue remodeling and occurs when an external force is applied to a tooth. The direct effect of such force is represented by a deformation or strain—sometimes infinitesimal—at the level of the tooth and its surrounding support tissues: the dentoalveolar ligaments and the alveolar bone. Cells within these tissues detect the strain and respond to the deformation of the extracellular matrix or to their own deformation, through the synthesis and secretion of various mediators, including cytokines and growth factors. Ultimately, this process leads to bone resorption on the pressure side of the moving tooth and bone apposition on the tension side, accompanied by the remodeling of the periodontal ligament [6].

The correct application of forces through orthodontic appliances enables optimal control of tooth movement and, consequently, the chances of unwanted side effects such as root resorption, pain, periodontal damage, pulpal degeneration and temporomandibular joint dysfunctions are reduced [1,7,8,9].

The factors that may influence the risk of complications of orthodontic treatment are represented by the type of appliance, force vectors and treatment duration, while patient-related factors which are relevant may be both biological and behavioral. Therefore, the various combinations among these categories may give rise to different forms of complications [10].

The correct application of force system influences the responses of biological tissues, inducing different responses in either a favorable or unfavorable manner. Precise biomechanical control is essential to avoid “round tripping” tooth movements, unnecessary increases in treatment duration and deterioration of the dentoalveolar tissues. When the system of forces and moments is incorrectly applied, with respect to duration, direction, magnitude and point of application, the outcomes of orthodontic treatment become unpredictable [11].

The physical behavior of tooth movement following the application of orthodontic force is governed by Isaac Newton’s laws of mechanics [12]. Among the three laws formulated by Newton concerning the interaction between forces acting upon a body and the motion of that body, the law of action and reaction (Newton’s Third Law) holds clinical relevance in orthodontics. In accordance with this law, once a force is applied to a single tooth, a group of teeth, or an entire dental arch, the supporting structure will respond with a force of equal magnitude and identical direction but opposite sense [13]. The laws of mechanics governing forces and movement within the orthodontic context are related to the material properties of appliance components, including stress, strain, stiffness, elasticity, and the elastic limit of archwires. Concepts such as moments and couples, the center of resistance and the center of rotation and the moment-to-force ratio are fundamental to understanding and controlling tooth movements. This foundational knowledge of the physical principles underlying orthodontics enables the clinician to design appliances and plan treatment protocols that will yield optimal outcomes [13].

To assure optimal control of tooth movement, the application of a well-designed force system is required. This system includes essential components such as orthodontic archwires, brackets and ligatures [14].

Knowledge of the responses of the tooth and its supporting structures during orthodontic treatment remains incomplete, as the histological techniques currently available can provide only limited information regarding the behavior of fundamental substance in cells, blood, and tissue fluids. A thorough understanding of several fundamental biological and mechanical concepts is therefore necessary to obtain comprehensive knowledge of clinical orthodontics [15]. The finite element method (FEM) is a numerical analysis approach characterized by a series of computational procedures involving the construction of a physical model, its subdivision into discrete smaller units termed “elements,” followed by the definition of material properties and the interconnecting relationships between elements [16]. It represents a valuable method for examining stress and strain distributions and for resolving complex structural problems in clinical applications, offering the advantages of avoiding ethical constraints while requiring considerably less time and lower costs compared to conventional experimental approaches [17,18].

In orthodontics, this method has been employed to evaluate the properties of craniofacial components and particularly to investigate stress distribution within the bone and periodontal ligament during various types of tooth movement [19,20]. The majority of studies have focused on the distalization [20,21,22] and mesialization [23,24] of posterior teeth, as well as on approaches for the orthodontic traction of impacted canines onto the dental arch [25]. Furthermore, this method has been utilized to analyze stress distribution within various components of orthodontic appliances, including archwires and temporary anchorage devices [26,27,28,29], as well as to guide the design of orthodontic appliances—encompassing the shape, length, positioning, and effect on adjacent structures of palatal expansion devices [30,31].

The objectives of the present in silico experimental study were to determine the forces generated within a fixed orthodontic treatment system comprising brackets and three types of orthodontic archwires and to evaluate the stress—in terms of tensions, displacements, and deformations—within the orthodontic system and at the level of the DMS for each of the three archwire types. Subsequently, multiple degrees of infraocclusion and linguoversion were simulated at the level of two teeth to determine the resulting stress distributions within the orthodontic systems.

## 2. Materials and Methods

The conduct of the present study encompassed several successive stages:

1. Virtual modeling of the DMS;
2. Modeling of the orthodontic system;
3. Determination of the forces generated within the orthodontic system;
4. Determination of the stress induced by orthodontic forces within the structures of the DMS.

### 2.1. Software Employed

To simulate mechanical behavior using FEM, several software applications were employed throughout this study.

AutoCAD 2022 was used for both two-dimensional (2D) drafting and three-dimensional (3D) modeling. This program integrates Direct Engineering techniques and computer-aided design (CAD) principles (Autodesk, Inc., 221 SE Ankeny Street, Portland, OR, USA). The Microsoft Office 2022 (Microsoft Corporation, Redmont, One Microsoft Way, 1, WA, USA) suite contributed to the generation of graphs and diagrams, the drafting of textual content, the organization of data and the automation of calculations. InVesalius 3.1.1 (CTI, Campinas, Brazil), an open-source software platform dedicated to medical scientific research, enabled the reconstruction of 3D structures of various human body tissues based on different grayscale values which are attributed to variations in tissue density, generating geometric structures in the form of point clouds [32]. Geomagic 2019 (3D Systems, Rock Hill, SC, USA), a software operating through techniques and methods specific to Reverse Engineering, was employed to convert the primary point cloud geometry into virtual solid bodies [33]. SolidWorks 2022 (Dassault Systèmes, Vélizy-Villacoublay, France), a CAD program utilizing Direct Engineering techniques and methods, facilitated the creation of virtual solid bodies, the generation of components with complex geometry, the assembly of components and the integration of orthodontic appliance elements, including archwires and brackets. The analysis of the mechanical behavior of the models was conducted in ANSYS Workbench 2019 (Ansys, Inc., Canonsburg, PA, USA) through the application of FEM, yielding simulations and results presented in the form of maps, diagrams and graphs [34,35].

### 2.2. Hardware Systems Employed

Computerized data analysis and the generation of graphs and diagrams were carried out using several desktop computers equipped with 8 GB of RAM and an Intel Core i3 processor operating at a frequency of 3.7 GHz. Additionally, a Lenovo laptop computer with 16 GB of RAM and an Intel Core i5 processor running at a frequency of 2.6 GHz was employed. Three-dimensional modeling and FEM simulations were performed on a Hewlett–Packard graphics workstation. For the analysis of 3D models and the interpretation of selected results, a Legamaster smart board (Legamaster International B.V., Lochem, Gelderland, The Netherlands), an Optoma video projector (Optoma, Coretronic Corporation, New Taipei, New Taipei City, Taiwan), and a desktop computer equipped with an Intel Core i3 processor (Intel Corporation, Santa Clara, CA, USA) were used.

### 2.3. Methods Employed

In the conduct of this study, the following methods were employed:

- Medical imaging methods, enabling the visualization of DMS structures;
- Methods of the Strength of Materials and the Theory of Elasticity, through which forces, deformations, displacements and stresses can be determined across a variety of conditions, applicable to both continuous and discontinuous media [36];
- Direct Engineering techniques and methods, in conjunction with CAD software tools, facilitating the 3D modeling of components and multi-body systems such as orthodontic archwires and bracket elements [37];
- Reverse Engineering methods, employed primarily in 3D scanning, enabled the acquisition of the 3D geometry of the DMS obtained from the patient through Cone Beam Computed Tomography (CBCT) [36];
- FEM involves the subdivision of the analyzed systems into smaller volumes, referred to as finite elements, which are subjected to various constraints or boundary conditions, fields, or loading systems. Finite elements are geometric bodies which possess faces, edges, and nodes. The mathematical formulas that maintain these finite elements within a virtual body are designated as constraint equations. The loading system, as well as the action of various fields, is expressed through mathematical equations containing complex derivatives and differentials. The parameters to be calculated are represented by a set of unknowns which can be found at the nodes of the finite elements. Therefore, there is a system of differential equations comprising *n* equations and *n* unknowns which appears to be a determinate compatible system. However, there are no generalized algorithms that permit the resolution of such a system of equations, except for some cases. FEM introduces an algorithm that transforms these differential equations into linear equations through successive iterations. The resulting system can subsequently be solved by means of techniques based on determinants and matrices. A simulation employing FEM enables the generation of result maps, graphs, and diagrams [38,39,40,41,42].

### 2.4. Acquisition of the Virtual Model of the DMS

**Figure 1:**
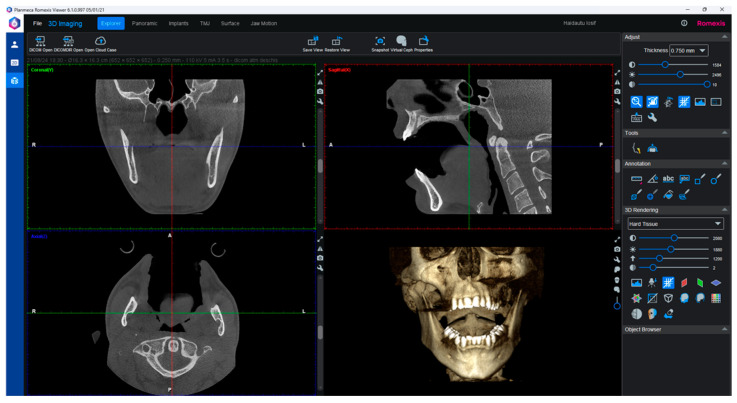
CBCT images of patient.

**Figure 2:**
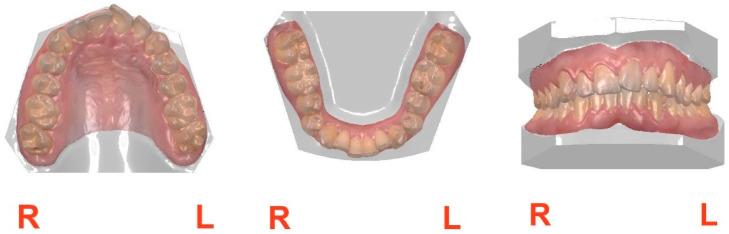
Study model of patient (R—Right; L—Left).

To perform a FEM analysis of craniofacial structures, the acquisition of precise geometric models is of paramount importance. Accordingly, a set of tomographic images obtained by means of computed tomography was utilized (Figure 1), accompanied by 3D images of both dental arches. Additionally, .stl format files of both arches were automatically generated, rendering them suitable for 3D printing (Figure 2). These investigations were conducted for a patient presenting with Angle Class I malocclusion and dento-alveolar disharmony with crowding. The research was approved by the Ethics Commission of the University of Medicine and Pharmacy of Craiova, No 253/6 November 2023. Informed consent was additionally obtained from the patient.

**Figure 3:**
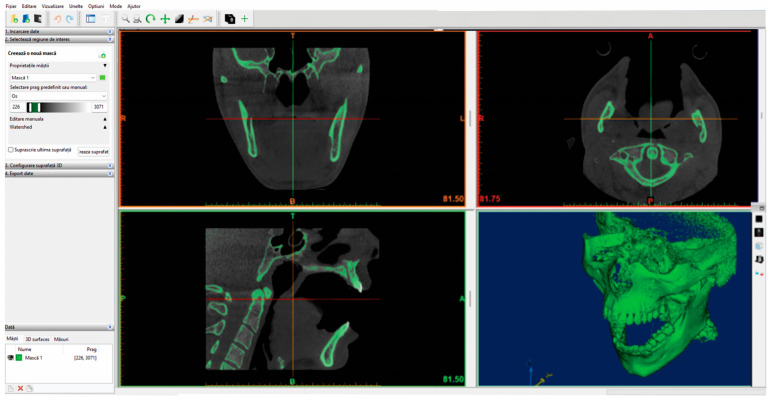
InVesalius software interface. Application of enamel filter for dental enamel.

**Figure 4:**
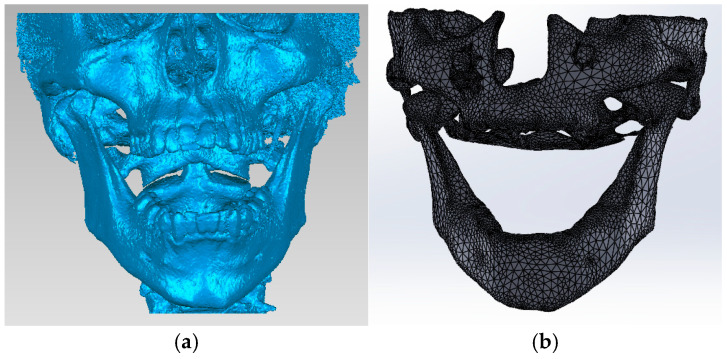
(**a**) Primary geometry of analyzed model in Geomagic. (**b**) Finalization stage of osseous components.

The patient’s CBCT tomographic dataset was imported into InVesalius software. Figure 3 illustrates the program interface following the uploading of the tomographic images and the application of the enamel filter (adult), while the primary geometry represented as a point cloud is presented in Figure 4.

#### 2.4.1. Three-Dimensional Modeling of Osseous Components

Given the fact that the grayscale values of dental tissues and osseous components are relatively similar, due to their comparable densities, the model was imported into Geomagic software for the execution of primary editing stages. Geomagic converted the geometry into spatial triangular surfaces, with the model initially comprising 8,193,730 such surfaces. Supplementary osseous components, including the cervical vertebrae, as well as the virtual dentition, were removed through dedicated operations. Subsequently, various techniques were applied for gap filling, the elimination of non-conforming surfaces and the reduction in the total number of elemental surfaces. The resulting model, presented in Figure 4b, was ultimately imported into SolidWorks and converted into virtual solid bodies.

#### 2.4.2. Three-Dimensional Modeling of the Dentition

**Figure 5:**
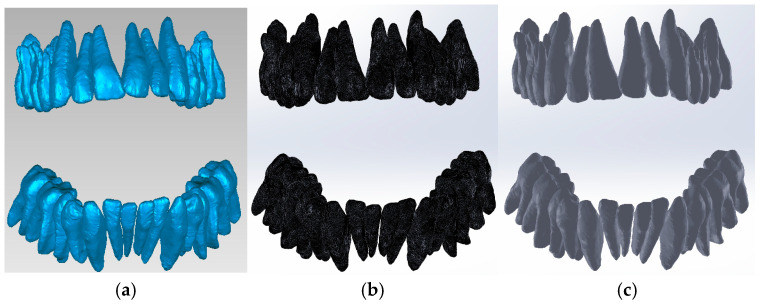
(**a**) Final model of dental arches—arch model in Geomagic. (**b**) Arch model in SolidWorks, first visualization mode. (**c**) Arch model in SolidWorks, second visualization mode.

The dental arches were identified through the application of the dental enamel filter. Elements belonging to the osseous components were subsequently removed through the application of several dedicated elimination operations. The resulting model of the dental arches was ultimately imported into SolidWorks and converted into virtual solid bodies (Figure 5).

#### 2.4.3. Three-Dimensional Modeling of the Periodontal Ligaments

**Figure 6:**
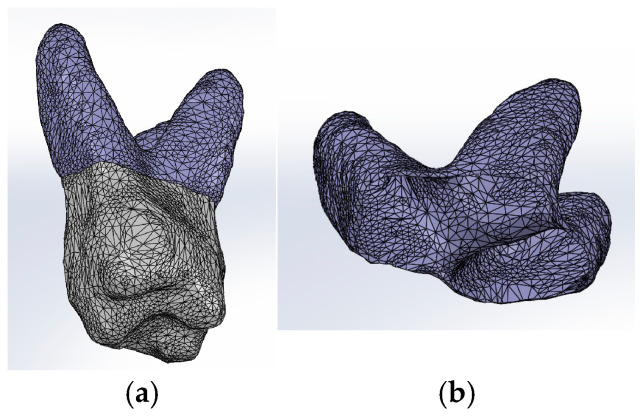
(**a**) Molar model with its corresponding ligament. (**b**) Ligament model.

To define the corresponding models for each periodontal ligament, each individual tooth model was imported into Geomagic. Offset surface definition techniques were applied to each individual model across its entire geometry, utilizing a distance of 0.2 mm. The coronal portion was subsequently removed from the resulting model and the obtained surface was closed with planar surfaces. Then, the model was exported into SolidWorks. The tooth model and the primary periodontal ligament model were sequentially loaded into the SolidWorks Assembly module. By means of common reference planes, the two models were brought into alignment. The final periodontal ligament model (Figure 6) was subsequently obtained by performing volumetric subtraction via the Cavity command.

#### 2.4.4. Three-Dimensional Modeling of the DMS

**Figure 7:**
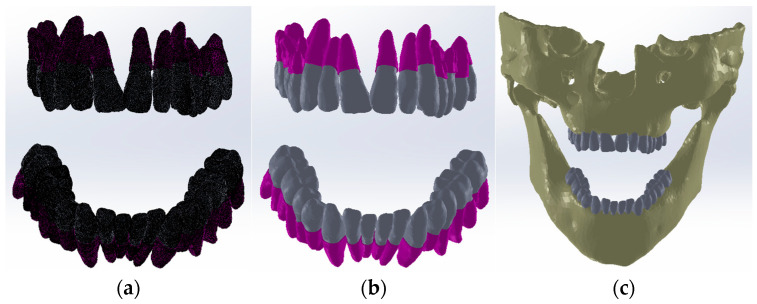
(**a**) Virtual models of dental arches; (**b**) periodontal ligaments; (**c**) osseous components.

Following the modeling of the osseous components of the DMS, along with the dental arches and their intra-alveolar components, the complete DMS model was obtained. To generate the dental alveoli within the two alveolar processes, a volumetric subtraction was performed between the two osseous components and the periodontal ligaments, followed by a further subtraction between the maxilla, mandible, and the dental arch model, by applying the Cavity command twice. The same methodology was applied to all teeth, and the result is represented in Figure 7. The resulting model incorporates the two osseous components (maxilla and mandible), the complete dental arches, and the periodontal ligaments (highlighted in magenta).

### 2.5. Obtaining the Virtual Model of the Orthodontic Appliance Components

#### 2.5.1. Three-Dimensional Modeling of Bracket Components

The bracket and tube components utilized in this study are Mini Prevail, with MBT prescription and a 0.022-inch slot. The dimensions of the brackets and tubes were the same as those used in the reference work of Bennett [43].

**Figure 8:**
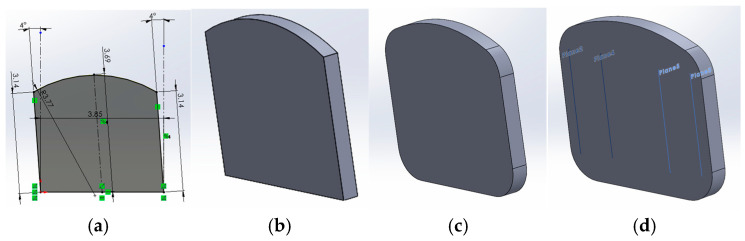
(**a**) Initial sketch of bracket element; (**b**) base feature of bracket component; (**c**) additional fillet features; (**d**) definition of reference planes.

The bracket components were modeled using Direct Engineering techniques, as well as principles of CAD. For the modeling of the bracket component corresponding to the upper right central incisor, an initial sketch was created in one of the reference planes, as illustrated in Figure 8a.

**Figure 9:**
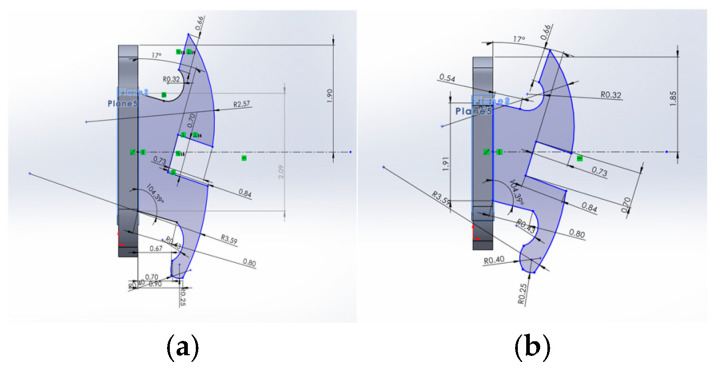
(**a**) Sketch drawn in first reference plane; (**b**) in second reference plane (original captures from SolidWorks).

Using this initial sketch, the basic shape of the component was obtained (Figure 8b), to which auxiliary features such as fillets were added (Figure 8c). Four parallel reference planes were subsequently defined, as illustrated in Figure 8d. In the first reference plane, the sketch presented in Figure 9a was created, while in the second reference plane, the sketch shown in Figure 9b was developed.

**Figure 10:**
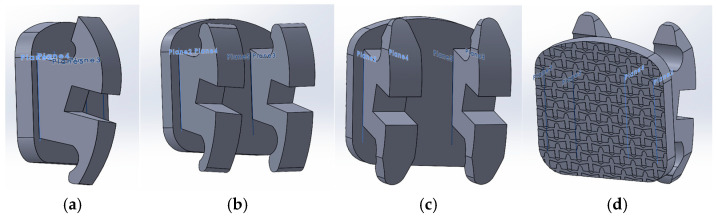
(**a**) First Loft feature; (**b**) second Loft feature; (**c**) bracket for right central incisor— slot and wings. (**d**) Bracket for right central incisor—base.

Using these two sketches, a Loft feature was defined, as illustrated in Figure 10a. Subsequently, two similar sketches were drawn in the remaining two reference planes, yielding a second Loft feature, as presented in Figure 10b.

**Figure 11:**
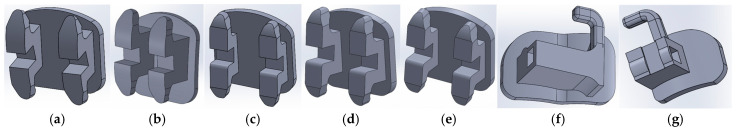
Models of the bracket elements for the teeth of quadrant I: (**a**) central incisor; (**b**) lateral incisor; (**c**) canine; (**d**) first premolar; (**e**) second premolar; (**f**) first molar; (**g**) second molar.

**Figure 12:**
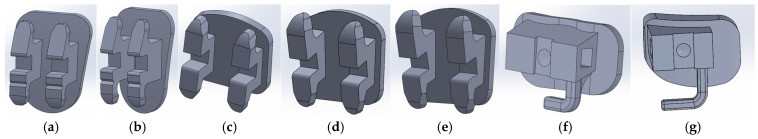
Models of the bracket elements for the teeth of quadrant IV: (**a**) central incisor; (**b**) lateral incisor; (**c**) canine; (**d**) first premolar; (**e**) second premolar; (**f**) first molar; (**g**) second molar.

Similarly, several additional features corresponding to the bracket base were incorporated, and the final model of the bracket component for the right central incisor was obtained (Figure 10c,d). Employing analogous modeling commands, 3D models of the bracket components were generated for the teeth of quadrants 1 (Figure 11) and 4 (Figure 12). Through the application of CAD mirroring techniques, the bracket elements for the teeth of quadrants 2 and 3 were subsequently derived.

#### 2.5.2. Three-Dimensional Modeling of the Orthodontic Archwire

**Figure 13:**
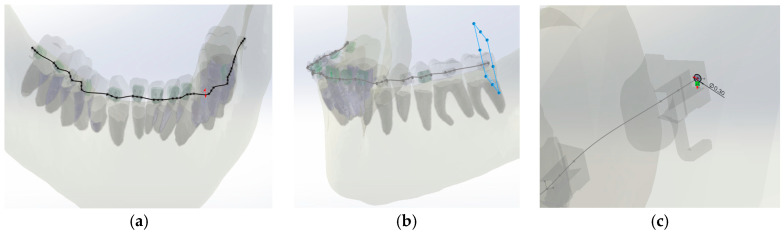
(**a**) The definition of a Spline curve on the bracket elements at the level of the mandible. (**b**) The definition of a reference plane. (**c**) Sketching of a circle within the reference plane.

In order to define the orthodontic archwire, three spatial reference points were plotted for each bracket component. Subsequently, a spatial Spline curve passing through all reference points on the bracket elements was drawn (Figure 13a).

A reference plane perpendicular to the spatial Spline curve was defined at one of its endpoints (Figure 13b). Within this plane, a circle with a diameter of 0.012″ was sketched (Figure 13c). Using this circle as the Profile and the spatial Spline curve as the Path, the model of the lower orthodontic archwire was defined by means of the Sweep feature. Employing analogous CAD techniques, the model of the upper orthodontic archwire was subsequently obtained.

To obtain the undeformed model of the lower orthodontic archwire, a 2D scan of the archwire was imported into AutoCAD and scaled to a 1:1 ratio. The curve defining this appliance was drawn over the scan and subsequently imported into SolidWorks, where it was employed as the guiding Path for the Sweep feature. The functional length of the archwire was determined by measuring the deformed archwire curve within SolidWorks. The undeformed archwire was trimmed to the functional length through virtual cutting, thereby yielding the final model of the undeformed lower archwire. An analogous procedure was followed for obtaining the undeformed upper archwire.

**Figure 14:**
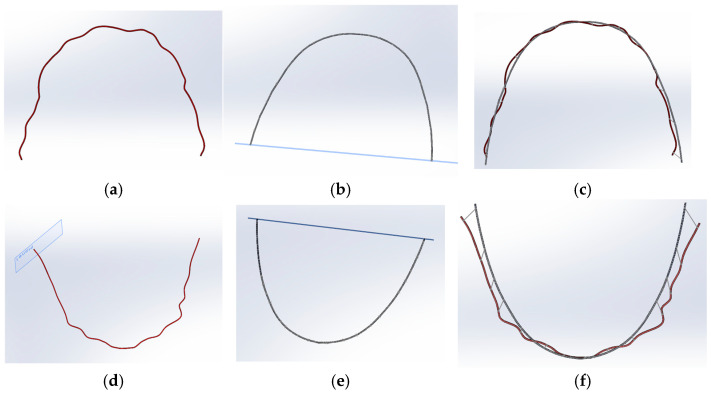
(**a**) A model of the lower orthodontic archwire; (**b**) the final model of the undeformed lower archwire; (**c**) a drawing of straight-line segments for the archwires corresponding to the mandible; (**d**) a model of the upper orthodontic archwire; (**e**) the final model of the undeformed upper archwire; (**f**) a drawing of straight-line segments for the archwires corresponding to the mandibular and maxillary arches.

The two models of the deformed and undeformed archwires for the mandible were loaded and superimposed within the SolidWorks Assembly module. Points corresponding to the bracket components were added for each tooth of the mandibular arch. These points were connected by straight-line segments drawn between the two archwires, thereby representing the archwire deformations. An identical procedure was applied for the upper orthodontic archwire (Figure 14).

#### 2.5.3. Obtaining the Model of the Orthodontic Adhesive

**Figure 15:**
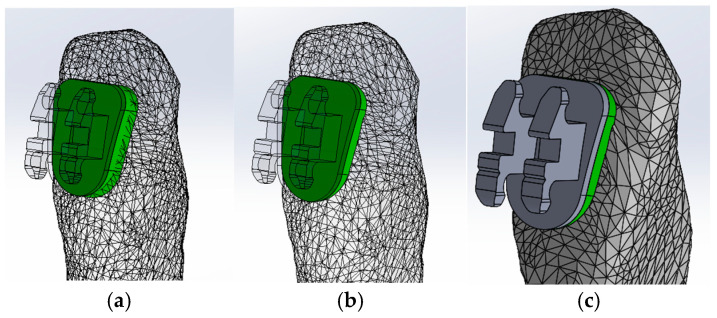
Obtaining the orthodontic adhesive model. (**a**) Editing of the adhesive model within the assembly context; (**b**) application of volumetric subtraction to the adhesive model; (**c**) the final model comprising the bracket element, adhesive, and tooth.

Using methods and techniques specific to Direct Engineering, an orthodontic adhesive model (depicted in green) was defined for each bracket component. The models of the bracket elements with the orthodontic adhesive were positioned on the tooth, with a region of volumetric interference being present. To obtain the accurate adhesive model, volumetric subtraction techniques were applied between the adhesive model and the tooth model (Figure 15).

### 2.6. Equations from Material Strength and Elasticity Theory Used to Determine the Forces Produced by Orthodontic Archwire

**Figure 16:**
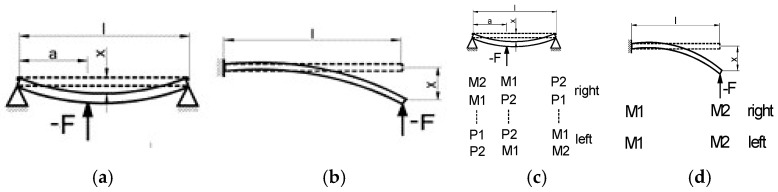
Modeling of bars subjected to reaction forces. (**a**) A recessed bar at both ends; (**b**) a bar with a free end; (**c**) application of the recessed bar pattern at both ends; (**d**) application of the model of the bar with a free end.

It is known that the forces in an orthodontic system arise as a result of the comeback of the deformed archwire to its initial, undeformed state. In the Strength of Materials and in the Theory of Elasticity, an algorithm for calculating bars with a constant cross-section on which forces act, has been developed. Two situations are known that can mathematically model the elastic behavor of an orthodontic archwire (Figure 16).

The first model can be applied on groups of three adjacent teeth, as can be seen in Figure 16a,c, where M—molar (M1-first molar; M2-second molar) and P—premolar (P1-first premolar; P2-second premolar).

The bar model that has a free end is applied to marginal teeth (Figure 16b,d) as a second molar (M2).

It is also known that force has the following general formula:

(1) F=k·x

where:

F—reaction force;
x—elastic deformation;
k—a constant that depends on the geometry of the bar, material, the position of the force on the bar.

For the model with the recessed bar at both ends, the constant k is as follows:

(2) $$k=\frac{9\;\sqrt{3}\cdot l\cdot E\cdot I}{a\cdot {\left(l^{2}-a^{2}\right)}^{3/2}}$$

where:

l—the length of the bar;
E—the modulus of elasticity (Young’s modulus);
a—the distance at which the force acts;
I—the axial moment of inertia;

In the case of a bar with a round section:

(3) $$I=\frac{\pi \cdot d^{4}}{64}$$

where d is the diameter of the bar.

For the model with a bar with a free end, the constant k is as follows:

(4) $$k=\frac{3\cdot E\cdot I}{L^{3}}$$

## 3. Results

### 3.1. Evaluation of the Forces That Are Generated by Ni-Ti Archwires of Different Diameters (0.012″, 0.014″, 0.016″)

#### 3.1.1. Determination of Orthodontic Archwire Deformations of Each Tooth

**Figure 17:**
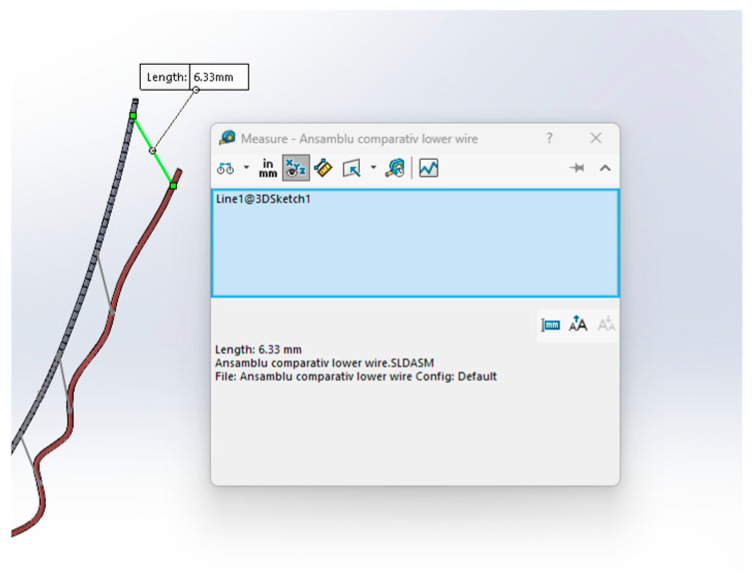
Deformation measurement for lower second molar.

In order to determine the forces, it was necessary to measure the deformations corresponding to each tooth on the overlapping models of orthodontic archwires (deformed archwire—undeformed archwire), as can be seen in Figure 17. Similarly, the x deformations were measured for all teeth (Table 1).

#### 3.1.2. Determination of Elastic Constant

Also, the distances of type **l** or **a** were also measured in the SolidWorks program. Three sizes of orthodontic archwires were used in the study, the conversion from imperial units to metric units being performed as follows: archwire with a diameter of 0.012″ (0.305 mm); archwire with a diameter of 0.014″ (0.356 mm); archwire with a diameter of 0.016″ (0.406 mm). The material of the v is nickel–titanium alloy, known as nitinol, and the modulus of elasticity is E = 34,500,000,000 Pa.

By applying Formulas (2) and (4), the values of the elastic constant k were obtained, both for the mandible and for the maxilla (Table 2).

The higher the thickness of the archwire, the higher the value of the elastic constant (k) in the orthodontic archwire (Table 3).

#### 3.1.3. Determination of the Forces Generated by Orthodontic Appliances

The forces generated with orthodontic appliances had different magnitudes, ranging from 0.007 N to 1.829 N, with most of them having a low magnitude. The average force was 0.390 N, and the median was 0.256 N (Table 4).

The computed forces should be interpreted as theoretical estimates under idealized conditions.

**Figure 18:**
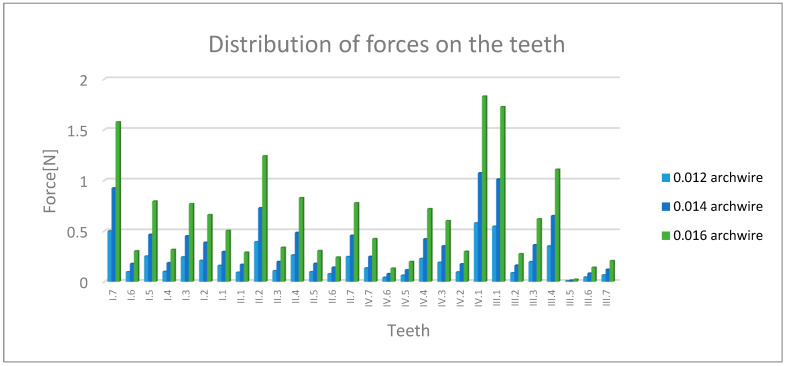
Force distribution.

A combined map showing the distribution of forces on each tooth was generated to facilitate direct visual comparison between the three archwire diameters (Figure 18).

Forces increased with archwire diameter across all simulated conditions; however, the distribution and relative magnitude of this increase varied non-uniformly across dental units, reflecting the influence of patient-specific arch geometry and local malposition configuration on the mechanical response of the system (Table 4 and Table 5).

The difference between the magnitude of the force generated by the 0.014″ diameter archwire and the 0.012″ diameter archwire averaged 0.17, and the median was 0.13. A larger difference was observed between the 0.016″ and 0.014″ diameter archwires. The mean was 0.25 and the median was 0.19 (Table 6).

**Figure 19:**
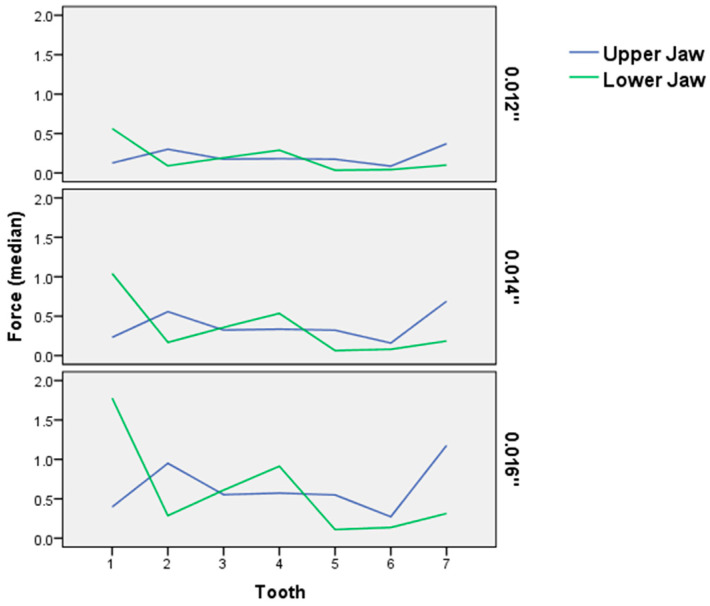
The magnitude of the forces depending on the arches and the archwires.

Analyzing the magnitude of the forces in relation to the tooth and the archwire used, a similar pattern of variation was observed at the level of both arches, but differences in amplitude in relation to the thickness of the archwire used were found (Figure 19).

For the case used, in the maxilla, the greatest forces were observed at the lateral incisor and second molar and the lowest at the first molar. For the mandible, the greatest forces were located at the central incisor and first premolar.

### 3.2. Analysis of Displacements, Deformations and Stresses and at the Level of DMS for Ni-Ti Orthodontic Archwires of Different Diameters (0.012″, 0.014″, 0.016″)

The present model includes several simplifying assumptions:

- The orthodontic archwire was replaced by an equivalent force system rather than being explicitly modeled;
- Friction at the bracket–archwire interface was neglected;
- NiTi behavior was approximated using a linear elastic model;
- The periodontal ligament was modeled as a homogeneous linear elastic material;
- The analysis was based on a single patient-specific geometry.

These assumptions were adopted to allow for a controlled evaluation of force transmission within the system.

The three-dimensional model of the dento-maxillary system, including teeth, periodontal ligament and alveolar bone, was imported into the FEM environment.

Boundary conditions were defined by constraining the basal regions of the maxilla and mandible to prevent rigid body motion. The loading conditions consisted of the force system derived from the analytical model, applied to the bracket elements in directions corresponding to archwire deformation.

The analysis was performed under the assumption of linear elastic material behavior for all components.

**Figure 20:**
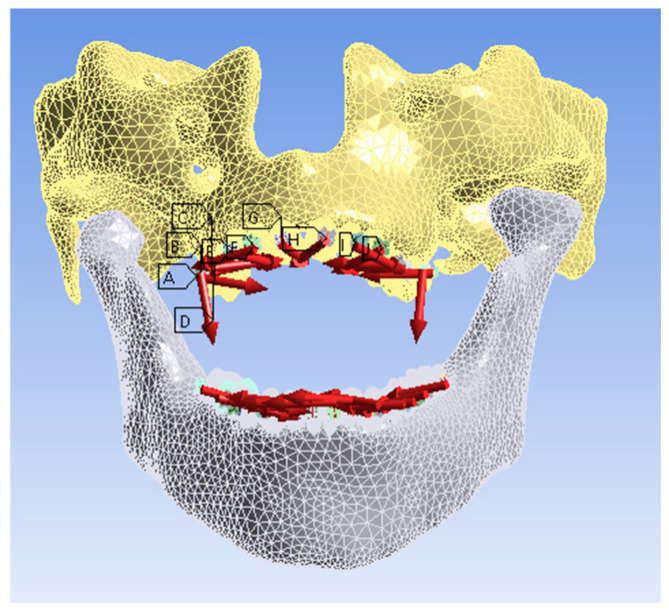
The system of forces acting on the orthodontic system.

The resulting model was loaded in Ansys Workbench [44], and the orthodontic archwires were suppressed and replaced with force systems. This system has been upgraded with the corresponding forces for orthodontic archwires with diameters of 0.012″, 0.014″ and 0.016″ (Figure 20). The model was divided into finite elements, obtaining 515,617 nodes and 250,905 tetrahedron elements [45,46]. The capital letters that appear in the image indicate how Ansys organizes its forces on the geometric model.

The orthodontic archwire was not explicitly modeled in the FEM simulation. Instead, it was replaced by an equivalent system of forces applied at the bracket level, derived from analytically calculated forces.

Homogeneous isotropic material properties were adopted as a simplifying assumption used in preliminary orthodontic FEM studies [47].

In the Engineering Data module, the materials needed for the simulation were introduced, as well as their physical and mechanical properties, as can be seen in Table 7 [48,49,50,51,52].

A sensitivity analysis was performed for the PDL modulus values for the most loaded biomechanical model when the orthodontic archwire has a diameter of 0.016 inches. Thus, the value of this constant was varied for 620 MPa, 62 MPa and 6.2 MPa. The values obtained are highlighted in Table 8.

The sensitivity analysis demonstrated that the absolute values of stress, strain, and displacement depend on the assumed PDL stiffness. Reducing the Young’s modulus of the periodontal ligament increased the predicted displacement and strain values, while stress values remained comparatively stable. These findings indicate that the quantitative results are influenced by the selected PDL properties; however, the overall biomechanical response of the model remained qualitatively consistent across the tested stiffness range.

To ensure the reliability of the numerical results, a mesh convergence analysis was performed using three successive mesh densities containing 250,905, 325,342, and 886,244 tetrahedral elements. Maximum von Mises stress, maximum displacement and maximum strain were selected as convergence indicators. The results obtained for the three mesh densities are presented in Table 9. The relative variation between the medium and fine meshes was 2.55% for stress, 7.31% for displacement, and 2.39% for strain. A convergence criterion of 10% was adopted. Since all monitored quantities satisfied this criterion, the mesh containing 250,905 elements was considered sufficiently accurate and computationally efficient for the subsequent simulations.

The selected PDL stiffness value was adopted from previously published FEM studies employing simplified linear elastic assumptions [53,54,55].

The stress analysis of virtual teeth using FEM included the following parameters:

- State of displacement: provides information on the variation in the positions of the nodes of the finite elements and is expressed in meters (m);
- State of deformation: according to the von Mises criterion of the mechanical system, it shows the elongation of the finite elements relative to the unit of length and is expressed in meter by meter (m/m);
- Stress state: obtained by the von Mises algorithm that provides information on the charge of finite elements by relating the force to the surface and is expressed in Pascals (1 Pa = 1 N/m^2^).

**Figure 21:**
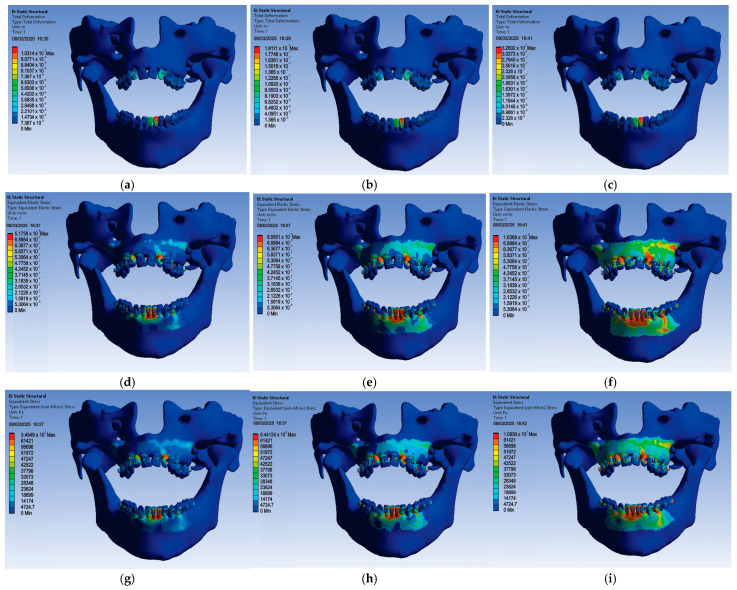
Displacement maps corresponding to orthodontic archwires with a diameter of A—0.012, B—0.014, and C—0.016 inch (**a**–**c**); deformations maps corresponding to orthodontic archwires with a diameter of A—0.012, B—0.014, and C—0.016 inch (**d**–**f**); stress maps corresponding to orthodontic archwires with a diameter of A—0.012, B—0.014, and C—0.016 inch (**g**–**i**).

Maps of the results were determined and were analyzed by using comparative charts (Figure 21).

**Figure 22:**
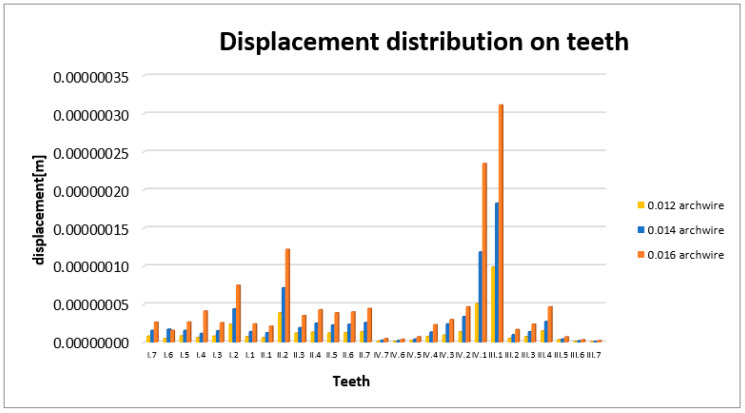
Displacement distribution diagram.

**Figure 23:**
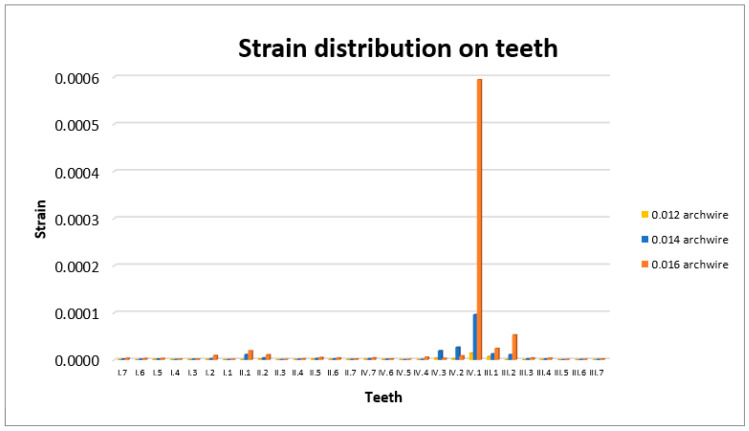
Strain distribution diagram.

**Figure 24:**
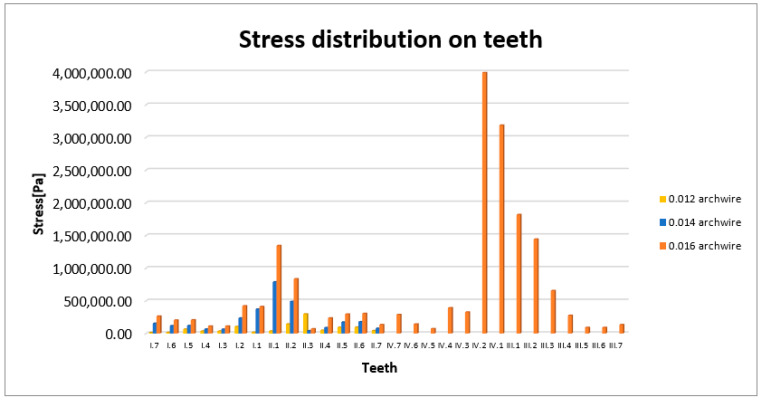
Stress distribution diagram.

Three combined maps showing the distribution of displacement (Figure 22), strain (Figure 23) and stress (Figure 24) on each tooth were generated to facilitate direct visual comparison between the three archwire diameters.

In the present study, the forces generated by the orthodontic archwires were determined using an analytical approach based on measured archwire deformations.

The orthodontic archwire was not explicitly modeled as a solid component within the finite element (FEM) simulation. Instead, it was replaced by an equivalent system of forces applied at the level of the bracket elements. These forces were derived from the deformation of the archwire, obtained by superimposing the deformed and undeformed configurations in the CAD environment.

The analytical model employed a simplified linear elastic formulation (F = kx), where the elastic constant k was determined based on the geometric and material parameters of the archwire. A Young’s modulus of 34.5 GPa was adopted for NiTi. The elastic modulus of 34.5 GPa is consistent with published data for superelastic NiTi archwires. Sabbagh et al. [56] report a value of 35 GPa for NiTi, and Uysal et al. [57] document the martensitic phase modulus in the range of 31–35 GPa.

The calculated forces were subsequently introduced into the FEM model as concentrated loads applied at the bracket level, corresponding to each tooth position.

The analysis of the mechanical parameters obtained by FEM—stresses, displacements and deformations (Figure 21)—reveals a consistent pattern of distribution of mechanical stresses within the fixed orthodontic system. It can be observed that high stress values are concentrated on the bracket components and on certain teeth, while being less pronounced in the orthodontic adhesive.

Significant variations were observed depending on the dental arch, the area analyzed and the type of archwire used.

From the perspective of the distribution on dental arches, the mandible recorded higher values for all mechanical parameters evaluated. The average mandibular tensions (491,066 Pa) exceeded by more than 50% the values of the maxillary tensions (213,722 Pa), a notable difference especially at the level of the maximum values (3,989,800 Pa compared to 1,344,600 Pa). A similar pattern was identified for displacements (0.0338 μm vs. 0.0359 μm) and deformations (23.746 με vs. 2.664 με), the latter showing the most marked discrepancy between the two arches (Table 10).

From the perspective of the topographic distribution, the canine–canine area was constantly distinguished by higher values compared to the premolar–molar area for all the evaluated parameters. The mean stresses recorded in the frontal region (666,025 Pa) were approximately 5.7 times higher compared to the posterior area (117,171 Pa), while the average deformations showed an even greater difference (28.642 με compared to 1.627 με), and the displacements followed the same trend (0.0502 μm compared to 0.0146 μm). The concentration of mechanical stresses in the frontal region, also supported by the maximum absolute values of stresses (3,989,800 Pa) and deformations (594.010 με) recorded in this area, can be correlated with the more pronounced deformation of the orthodontic archwire in the canine–canine segment, confirming that the lower intercanine area represents the critical point of the orthodontic system analyzed (Table 11).

Regarding the influence of the type of archwire, the results consistently confirm the existence of a directly proportional relationship between the size of the cross-section and the magnitude of the mechanical stresses generated in the system. The 0.012″ archwie produced the lowest values for all the analyzed parameters (mean stresses 119,323 Pa, average displacements 0.0141 μm, average deformations 1.561 με), followed by the 0.014″ arc (299,964 Pa, 0.0276 μm, and 7.596 με respectively), while the 0.016″ arc generated the highest values (637,895 Pa, 0.0478 μm, and 30.457 με respectively), with absolute maximums of 3,989,800 Pa, 0.3113 μm and 594.010 με (Table 12).

### 3.3. Evaluation of the Forces Resulting from the Displacement of an Incisor of Quadrant III in Different Degrees of Linguoposition of a Canine from Quadrant I in Different Degrees of Infraposition

#### 3.3.1. Evaluation of the Forces Resulting from the Displacement of an Incisor in Quadrant III in the Linguoposition

**Figure 25:**
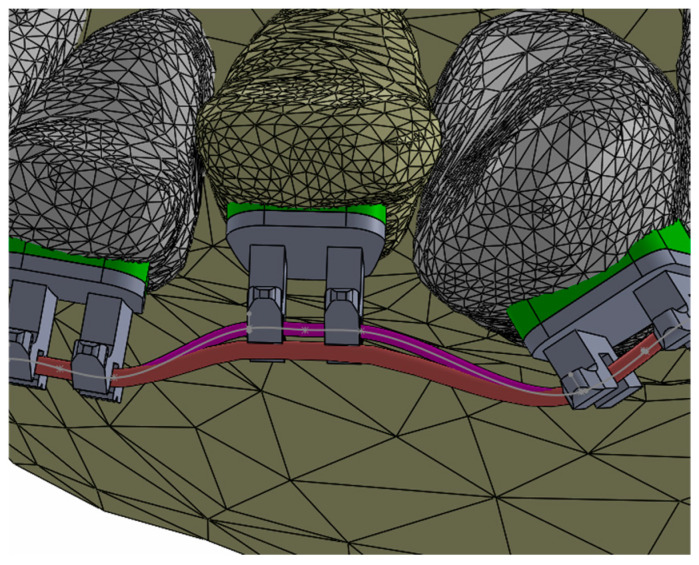
Defining a new orthodontic archwire.

The simulation process began by moving the incisor-adhesive bracket assembly by 0.5 mm towards the lingual part. This assembly was introduced into the model of the orthodontic system, where the old incisor was suppressed. The new orthodontic archwire (magenta color) was defined through the bracket component, as can be seen in Figure 25.

**Figure 26:**
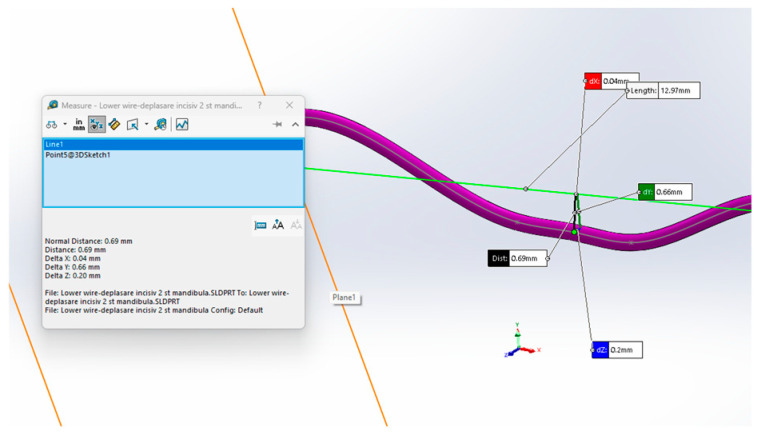
Elastic deformation measurement.

On this model, the elastic displacement was measured (Figure 26) and a value of 0.69 mm was obtained, as can be seen in Figure 24. By entering this value into the algorithm based on Equations (1)–(3), the value of the force F = 0.206714 N (corresponding to the archwire with a diameter of 0.012″) was obtained.

In order to assess the response of the orthodontic system to progressive deformities, the stress imposed at 0.5 mm was changed to 1 mm, resulting in a corresponding update of the archwire geometry. In this scenario, the elastic deformation measured was 1.16 mm, generating a force of 0.348 N (corresponding to the 0.012″ diameter spring).

In the final stage of the simulation, the incisor displacement was set to 1.5 mm, resulting in an elastic deformation of 1.63 mm. Entering these data into the implemented calculation algorithm provided a force value of 0.488 N (corresponding to the archwire with a diameter of 0.012″).

#### 3.3.2. Evaluation of the Forces Resulting from the Displacement of a Canine from Quadrant I in Infraposition

**Figure 27:**
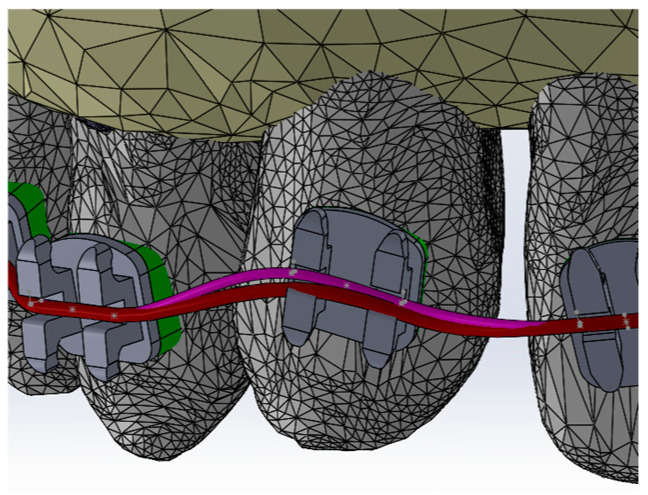
Moving the canine 0.5 mm upwards.

The techniques previously applied to the canine in quadrant I were applied. Figure 27 shows the model for upward movement (infraposition) by 0.5 mm.

The elastic deformation of the orthodontic archwire was determined, obtaining a value of 1.87 mm. From the algorithm developed for the calculation of the force, its value was determined to be equal to 0.267 N (for a 0.012″ archwire).

Next, the shifting distance was changed to 1 mm. For this scenario, the virtual measurement indicated an elastic deformation of the orthodontic archwire of 2.23 mm, corresponding to a force of 0.318 N (corresponding to the arch diameter of 0.012″).

In the final stage, the canine moved by 1.5 mm. The elastic deformation recorded was 2.64 mm. The application of the calculation algorithm for this deformation value generated an elastic force of 0.377 N (corresponding to the archwire diameter of 0.012″).

**Figure 28:**
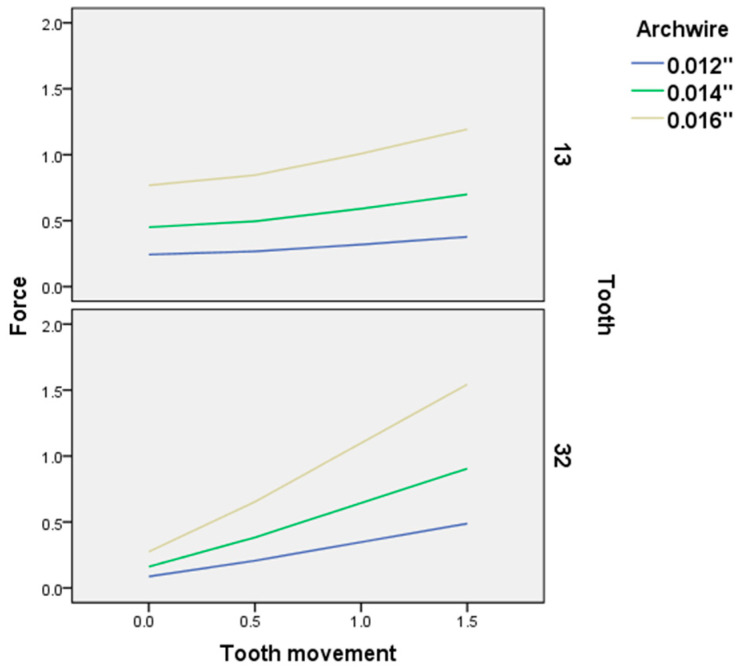
Magnitude of force as function of tooth displacement at teeth 32 and 13 for arch with diameters of 0.012″, 0.014″, 0.016″.

The same process was applied for archwires with diameters of 0.014″ and 0.016″ (Figure 28 and Table 13).

## 4. Discussions

In the last decade, the transfer of information from CT or CBCT images to virtual analysis environments has become an increasingly used operation in dental research. These processes involve the application of Reverse Engineering, Direct Engineering or FEM analysis techniques on a geometry almost identical to the clinical reality of the patient, in accordance with the requirements of contemporary dentistry.

FEM has been employed in orthodontics since 1973, being used since then in a growing number of studies analyzing stresses and deformations at the level of dento-alveolar structures, as well as in the validation of theories in the field of dental biomechanics, which investigate the reactions of alveolar tissue associated with dental displacement [58]. The convergence between biological and engineering sciences, facilitated by various recent technological developments, has led to a precise understanding of the properties of human tissues, different materials and techniques used in dentistry at the microbiological and ultrastructural level, with FEM being one of the most important research tools in this context [59].

The applications of this method in orthodontics cover a wide spectrum of biomechanical and clinical issues. FEM has been used for the analysis of stress distribution at the level of the adhesive bracket–tooth complex, demonstrating that the thickness and geometry of the periphery of the adhesive layer significantly influence the distribution of mechanical stresses at this level [60]. The method also allowed for the evaluation of stress distribution at the level of ceramic brackets, with studies demonstrating that stresses are usually concentrated at the edges and corners of ceramic brackets [58,59]. FEM is a valuable tool to compare the stress generated by orthodontic mini-implants made of various materials, but also to assess the stress generated at different degrees of insertion [61,62]. At the same time, it can facilitate the understanding of the mechanisms that determine dental displacement [63].

Other studies have investigated through FEM the mass retraction of the frontal group through sliding mechanics [64], demonstrating that when the hook is positioned between the lateral incisor and canine, mass retraction of the anterior segment is better controlled. Also, through this method, the mechanical stresses generated by conventional maxillary expansion and by implant support devices were compared [65]. In the present study, a set of CBCT images taken from a patient were processed and transformed into “point cloud” geometries. This “point cloud” was transformed, through techniques specific to Reverse Engineering, into a large number of triangular 3D surfaces, and then into 3D surface structures corresponding to each type of tissue.

Taking data from this system and applying algorithms specific to the Strength of Materials, the forces acting on each bracket component were calculated, due to the elasticity of the orthodontic archwires. These forces depend on the geometry of the deformed archwire, its diameter and the material of the orthodontic archwire (in this study, the material is the nickel–titanium alloy known as nitinol).

The biological response generated in the surrounding tissues, from which the orthodontic movement of the tooth results, depends on the type, magnitude, direction and duration of the force, as well as the age of the patient [15]. Among the complications frequently reported in the literature are apical external root resorption [66], tooth enamel demineralization [67], periodontal diseases [68], and allergic reactions to the materials used in the composition of fixed orthodontic appliances [69].

From a periodontal perspective, fixed orthodontic appliances act as plaque retention factors, making it difficult to achieve proper oral hygiene and thus favoring the onset of gingivitis, considered the most common short-term complication of orthodontic treatment at the periodontal level. In cases of chronic inflammation, the process can progress to fibrosis, clinically manifested by gingival hyperplasia or hypertrophy, with implications for aesthetics and periodontal health in the long term [70].

Mechanical complications—such as fracture of brackets or archwires, displacement of device components or improper use of the appliance by the patient—can compromise the results of treatment and generate additional adverse effects [10]. Reducing the incidence of these complications is conditional on compliance with clinical protocols, maintaining rigorous oral hygiene throughout treatment, and active collaboration between the patient and the orthodontist [1].

Under normal conditions, dental displacement is achieved through highly coordinated and efficient bone remodeling, which requires the interaction between two phenomena—bone apposition and resorption. Within seconds of applying force, the tooth changes position within the viscoelastic matrix of the periodontal ligament, resulting in compression of the ligament in some areas and its stretching or tension in others. While blood flow is reduced on the compression side, it is maintained or increased on the tension side. If the strength is maintained, the change in blood flow quickly influences, within a few minutes, the ratio of O_2_:CO_2_ in the chemical environment by releasing biologically active agents, such as prostaglandins and cytokines (interleukin (IL)-1β). These chemical mediators differentially influence cellular activity in the compression and tension areas of the periodontal ligament, favoring the triggering of bone resorption phenomena on the compression side and bone formation on the tension side [12].

From a physical point of view, dental movement is a process that is based on the principle of storing elastic energy and converting it into mechanical work, causing dental displacement.

For the first phase of orthodontic treatment, the leveling and alignment phases, orthodontists opt for a round archwire, which can be easily bent and will be replaced by rectangular archwires as treatment progresses. This sequence of archwires is important to obtain the lightest possible forces during the orthodontic treatment.

For years, the use of nickel–titanium alloy (NiTi) archwires has been preferred over the use of stainless steel archwires in the early stage of orthodontic treatment [71].

NiTi archwires have proven their clinical effectiveness in orthodontics due to their outstanding properties. Shape memory is the property of returning to the original shape after plastic deformation as a result of the reversible transformation from the austenitic to the martensitic phase. These archwires also exhibit low rigidity, so they exert small deactivation forces on the surface unit. The superelastic behavior of these archwires has proven to be suitable for the alignment and leveling stage, providing a constant force for tooth displacement [72]. As the orthodontic archwire tends to recover its original shape, it also displaces the dental structures behind it [14].

With each activation of the orthodontic appliance, energy is stored and then gradually released, thus controlling the transfer and distribution of the applied forces. To ensure optimal control of tooth movement, it is necessary to apply a well-designed force system [14].

The magnitude of the force is associated with varied cellular responses on the compression side. The force of high magnitude completely interrupts the blood flow by obliterating the blood vessels, which causes necrosis of the area served by the blood vessels. Due to its specific histological appearance, this area of necrosis has been referred to as the hyalination zone. In this compressed region of the periodontal ligament, osteoclastic differentiation does not occur. In contrast, the recruitment and differentiation of osteoclasts occur late from the adjacent medullary space that is unaffected. This process is responsible for “undermining resorption”, which removes the hard lamina next to the compressed periodontal ligament. The dental displacement following this process is slow, taking 7–14 days for the movement of the teeth to occur when a force of increased magnitude is applied [73]. In 1905, Carl Sandstedt’s research on dogs demonstrated that tooth movement is a process that involves bone resorption and apposition. He was the first to describe the glassy appearance of compressed tissue, resulting from hyalinization and which was associated with a temporary arrest of tooth movement [15].

In contrast, a low-magnitude force only causes a reduction in blood flow by partially blocking the blood vessels, which allows for the rapid recruitment of osteoclasts, either locally in the periodontal ligament or through blood flow. These osteoclasts remove the lamina dura through a process of “frontal resorption”. Therefore, dental displacement begins immediately afterwards, usually within 2 days of applying light force. Clinically, it is almost impossible to completely avoid blood vessel obliteration, so hyalinization always occurs to some extent. Thus, dental movement is the result of a combination of undermining resorption and frontal resorption [73].

Therefore, it is very important that during orthodontic treatment you achieve as light a force as possible. Tooth movement is effective at forces below 1N (Table 14) [74,75].

Regarding our study, some calculated force magnitudes exceeded commonly referenced orthodontic force ranges; however, these values should not be interpreted as direct clinical tooth-loading thresholds. Because the reported forces were obtained as equivalent bracket-level forces derived from local archwire deformation, they should not be interpreted as net whole-tooth forces. Consequently, direct comparison with clinically reported optimal force thresholds was not considered appropriate. The present results should instead be interpreted comparatively, highlighting how increasing archwire deformation and diameter affect the magnitude of the generated force system within the assumptions of the model.

The data obtained through finite element analysis indicate that bracket-level forces increase substantially across the dental arch with increasing archwire diameter and deformation magnitude. This pattern is most evident at dental units 31 and 41, which recorded the highest force values across all three archwire dimensions: for the 0.014″ archwire, forces of 1.01 N and 1.07 N were generated at deformations of 1.07 mm and 1.41 mm, respectively, while the 0.016″ archwire produced markedly higher values of 1.73 N and 1.83 N at the same dental units. Additional dental units showed notably elevated force magnitudes with the 0.016″ archwire, namely tooth 17 (deformation 1.07 mm, force 1.58 N), tooth 22 (deformation 2.76 mm, force 1.24 N) and tooth 34 (deformation 1.65 mm, force 1.11 N), confirming that progression to a larger archwire diameter substantially increases the number of dental units subjected to elevated mechanical loading within the assumptions of the present model. For the 0.012″ archwire, force magnitudes remained considerably lower across all dental units, with the highest values recorded at teeth 31 (0.55 N), 41 (0.58 N) and 17 (0.50 N).

The analysis of the elastic deformation of the orthodontic archwire according to the degree of dental malposition investigated (linguoposition of tooth 32 and infraposition of tooth 13) highlights progressive values for both dental units investigated.

At the incisor level, linguoposition displacement showed an approximately linear relationship with archwire deformation, ranging from 0.69 mm at 0.5 mm displacement to 1.16 mm at 1 mm and 1.63 mm at 1.5 mm. The 0.012″ archwire generated bracket-level forces of 0.21 N, 0.35 N and 0.49 N at 0.5, 1 and 1.5 mm displacement, respectively. The 0.014″ archwire produced forces of 0.38 N, 0.64 N and 0.90 N at the same displacement increments, while the 0.016″ archwire generated the highest values: 0.65 N, 1.10 N and 1.54 N at 0.5, 1 and 1.5 mm displacement, respectively. At the canine level, infraposition generated considerably greater archwire deformations than linguoposition at equivalent displacement magnitudes: 1.87 mm at 0.5 mm, 2.23 mm at 1 mm and 2.64 mm at 1.5 mm. The 0.012″ archwire generated forces of 0.27 N, 0.32 N and 0.38 N at 0.5, 1 and 1.5 mm displacement, respectively. The 0.014″ archwire produced forces of 0.50 N, 0.59 N and 0.70 N, while the 0.016″ archwire generated the highest mechanical demand: 0.85 N, 1.01 N and 1.19 N at 0.5, 1 and 1.5 mm displacement, respectively. These values must however be interpreted in conjunction with the real mechanical conditions at the archwire–bracket interface. The friction developed at this level, estimated in the literature to be 12% to more than 70% [76,77], acts as a dissipative factor, reducing the active force effectively transmitted to the tooth relative to the values calculated by the FEM model. Therefore, the forces obtained in the present study represent theoretical maximum values and the actual orthodontic force reaching the level of the periodontal ligament will be lower, depending on the tribological characteristics of the bracket–archwire system employed.

This perspective does not diminish the biomechanical relevance of the identified deformation–force correlation. Archwire deformations exceeding 1 mm are associated with substantially higher bracket-level forces, particularly for the 0.016″ archwire, which generated markedly elevated force values across a greater number of dental units. The 0.012″ and 0.014″ archwires generated considerably lower forces across all simulated conditions, supporting their preferential use in early alignment stages.

In most clinical scenarios, 0.016-inch nickel–titanium archwires demonstrate effective initial leveling and aligning performance [43]. Nevertheless, in cases involving severe malocclusions or requiring large deflections, the selection of the initial archwire should be approached with greater clinical judgment, as higher deflection demands may necessitate the use of smaller-diameter wires to ensure optimal force delivery and patient comfort.

The directly proportional progression of stresses, displacements and deformations from the 0.012″ archwire to the 0.016″ archwire confirms that the selection of the archwire constitutes an essential biomechanical decision, with direct implications both on the clinical efficiency of the treatment and on the risk of overloading the components of the orthodontic system. In this context, the gradual increase in the size of the archwires is essential from a biomechanical perspective, allowing the system to gradually adapt to increasing mechanical stresses.

Direct numerical comparison with published experimental data is constrained by fundamental methodological differences. Published in vitro studies employing force gauges or universal testing machines typically measure forces on isolated archwire segments in three-point or three-bracket bending configurations under standardized but simplified geometric conditions that do not replicate full-arch loading across a complete dentition with prescription-specific bracket angulations. Notwithstanding these differences, a comparison remains possible. Munir et al. reported that a 0.016″ superelastic NiTi archwire registered a maximum loading force of 3.8 N at 3.1 mm deflection in a three-point bending configuration, with the unloading force stabilizing between 2.0 N and 2.2 N from 2.5 mm to 0.5 mm deflection. The bracket-level force magnitudes predicted in the present model for the 0.016″ archwire are of a lower order than the experimentally measured values in Munir et al., which is consistent with the fact that the present model assumes frictionless bracket–archwire contact and applies forces at clinically moderate deflection magnitudes (0.5–1.5 mm) rather than at the large deflections used in standardized bending tests [78].

The originality of the present study resides in the systematic parametric quantification of the relationship between pre-existing dental malposition severity and archwire elastic deformation and its expression as differential mechanical loading at individual dental units across three archwire dimensions. This originality lies not in the analytical force formulation itself, which is based on established beam mechanics, but in its integration with a patient-specific full-arch computational framework incorporating MBT prescription-specific bracket geometry and individualized archwire deformation measurements. Bracket elements were modeled in accordance with the MBT prescription, incorporating torque, angulation and in-out values specific to each dental unit, in this way ensuring that the archwire–bracket mechanical interaction reflects a clinically standardized configuration rather than a geometric idealization.

The present study quantifies the elastic deformation of the orthodontic archwire and the bracket-level forces generated as a direct function of that deformation across varying degrees of dental malposition and archwire dimensions.

The principal contribution of the present study is the development of a hybrid analytical–finite element workflow that links patient-specific archwire deformation to the resulting distribution of stresses, strains and displacements within a standardized computational environment. The approach is intended to provide comparative biomechanical insight rather than direct prediction of clinical outcomes.

This approach also demonstrates that minor dental malpositions may induce archwire deformations sufficient to generate substantially elevated bracket-level forces, particularly when larger archwire diameters are employed. The results indicate that deformations exceeding approximately 1 mm are associated with a marked increase in force magnitude, particularly at the level of the mandibular incisors. Within the comparative framework of the present model, these findings support careful archwire selection at the outset of treatment and highlight the biomechanical rationale for graduated archwire sequencing in the presence of unresolved dental malpositioning.

The methodology of the present study, based on 3D reconstruction from CBCT images and FEM analysis, provides a robust platform for integrating artificial intelligence (AI) techniques into the later stages of research. The results of the present study confirm that the magnitude of orthodontic forces depends significantly on the diameter of the arch and the degree of dental malposition. Based on a sufficiently large dataset, these biomechanical relationships can be formalized by regression models or by gradient boosting algorithms (XGBoost, LightGBM), capable of predicting the forces generated by different dental arc malposition configurations. Such an approach would support the clinical decision on arc sequencing and contribute to the individualization of treatment protocols.

The FEM analysis, although rigorous, involves high computational costs, especially for models with complex geometries and a large number of nodes (in the present study, 515,617 nodes and 250,905 tetrahedron-like elements).

The present study investigated the mechanical behavior of fixed orthodontic systems by combining analytical force estimation with finite element analysis applied to a patient-specific geometry. Anatomical variability between patients, including differences in arch morphology, tooth inclination, and bone density, may substantially affect the resulting stress distribution and force transmission patterns.

The results confirm a direct relationship between archwire diameter and force magnitude, which is consistent with classical beam theory, where stiffness increases with the fourth power of the wire diameter. This trend was reflected in the increased stress, displacement and deformation observed in the FEM simulations. The central clinical question of the present study was whether a specific archwire deformation threshold can be identified beyond which mechanically relevant forces are generated.

However, several methodological aspects must be considered when interpreting these findings.

First, the orthodontic archwire was not explicitly modeled in the FEM simulation. Instead, it was replaced by an equivalent system of forces derived from analytical calculations based on archwire deformation. This approach allows for controlled application of forces but does not capture complex interactions at the bracket–wire interface.

Second, the analytical model employed a linear elastic approximation (F = kx) for NiTi archwires. In reality, NiTi exhibits superelastic behavior characterized by a nonlinear stress–strain response and a plateau region in which force remains relatively constant over a range of deformations. Therefore, the forces calculated in this study should be interpreted as approximate values under simplified conditions rather than exact representations of clinical behavior.

Third, friction between the archwire and bracket slot was not included in the model. It is well documented that frictional resistance can significantly reduce the effective force transmitted to the tooth. Consequently, the forces reported in this study represent upper-bound estimates. The loss of the applied force due to friction as reported in the literature ranges from 12% to more than 70% [76,77].

Another important limitation concerns the mechanical properties assigned to the periodontal ligament and bone. Although a constant elastic modulus was used, the PDL is known to exhibit highly nonlinear and viscoelastic behavior. Variations in its stiffness can significantly influence stress distribution and tooth displacement patterns. To assess the influence of PDL stiffness on model outputs, a sensitivity analysis was performed by varying the PDL Young’s modulus across a range of three orders of magnitude (6.2, 62 and 620 MPa) for the most mechanically loaded configuration. The sensitivity analysis further demonstrated that the absolute magnitudes of the predicted mechanical parameters depend on the assumed PDL stiffness. Therefore, the reported stress, strain and displacement values should be interpreted within the context of the selected material properties, whereas the comparative trends observed between loading conditions appear more robust.

The assumption of homogeneous isotropic bone properties represents a simplification adopted in preliminary FEM orthodontic studies. The virtual model of DMS does not differentiate compact from spongy bone, using the same mechanical properties for both types of bone in the calculations.

Cortical and trabecular bone exhibit heterogeneous mechanical behavior that may influence local stress propagation and displacement patterns. Consequently, the present results should be interpreted within the limitations of the simplified material model [47]. Since the trabecular and cortical bone domains cover a wide range of density, dividing the material model into sub-functions based on density ranges, known for both bone domains, is an option to better estimate the Young’s modulus. Therefore, Pinheiro et al. [79] created separated sub-functions for the cortical and trabecular domains. However, this approach poses a challenge of discontinuity in material properties at the interface of bone domains, leading to complications in mesh convergence in FE analysis. An alternative strategy is provided by Keyak et al. [80], who addressed this issue by considering the transitional domain between cortical and trabecular bone within a linear sub-function, which ensures continuity at the boundaries [81]. In future work, a heterogeneous material model based on continuous density-dependent sub-functions, similar to the approach proposed by Keyak et al., will be implemented to achieve a more physiologically accurate representation of bone mechanics while preserving continuity at the cortical–trabecular interface.

Furthermore, this study is based on a single patient-specific geometry. The results therefore represent deterministic outputs of a specific anatomical configuration and cannot be generalized to a broader population. For this reason, the findings should be interpreted as case-specific rather than population-based conclusions.

Consistent with these constraints, the finite element component of the present workflow should be regarded as an exploratory biomechanical tool intended to visualize relative stress and displacement patterns under controlled assumptions. The generated stress maps are therefore useful primarily for comparative interpretation within the model framework and should not be considered quantitative representations of in vivo tissue loading.

Despite these limitations, this study provides valuable insights into the relative influence of archwire diameter and tooth position on force transmission within the orthodontic system. The integration of patient-specific geometry with analytically derived force systems represents a useful framework for understanding biomechanical behavior under controlled conditions.

The principal contribution of the present study is the development of a hybrid analytical–finite element workflow that links patient-specific archwire deformation to the resulting distribution of stresses, strains and displacements within a standardized computational environment. The present study represents, to the best of our knowledge, one of the few FEM investigations to employ a full-arch model incorporating MBT prescription, with a specific bracket geometry with individualized torque, angulation and in–out values at each dental unit, in conjunction with the initial leveling and alignment archwires used during the first phase of treatment. The approach is intended to provide comparative biomechanical insight rather than direct prediction of clinical outcomes.

The absence of a directly equivalent computational benchmark is therefore an inherent consequence of the methodological originality of the present approach. Direct numerical comparison with published in vitro data is further constrained by fundamental methodological differences, as experimental studies typically measure forces on isolated archwire segments under simplified geometric conditions that do not replicate full-arch loading with brackets that incorporate MBT prescription. Previous FEM studies have employed equivalent force-based loading systems instead of explicitly modeling the archwire geometry, supporting the methodological validity of the present computational framework [47,82].

Future research should aim to incorporate nonlinear constitutive models for NiTi, include frictional interactions at the bracket–wire interface and extend the analysis to multiple patient geometries in order to improve clinical relevance and generalizability.

## 5. Conclusions

Within the assumptions of the present model, larger archwire diameters were associated with higher calculated forces and increased stress, strain and displacement values. These observations should be interpreted as comparative biomechanical findings rather than direct clinical predictions.

The results indicate that smaller-diameter archwires generate lower force levels, while larger diameters produce higher mechanical responses.

Archwire elastic deformation, determined by pre-existing dental malposition geometry, constitutes a meaningful predictor of mechanical loading magnitude at individual dental units, with direct implications for archwire selection and sequencing The results demonstrate that even moderate degrees of malposition may induce deformations sufficient to generate substantially elevated bracket-level forces, particularly with larger archwire diameters, supporting careful archwire selection from the outset of treatment.

However, these findings must be interpreted considering the study limitations, including the use of a simplified analytical force model, the assumption of linear elastic material behavior for NiTi, the neglect of friction at the bracket–wire interface and the use of a single patient-specific geometry.

Therefore, the reported values should be regarded as theoretical estimates that provide comparative insights into orthodontic force systems rather than exact representations of clinical conditions.

## Figures and Tables

**Table 1:** Maximum deformations in the orthodontic arch.

Maximum deformations (x) in the orthodontic arch -[mm] in the upper teeth													
17	16	15	14	13	12	11	21	22	23	24	25	26	27
1.07	0.29	1.34	1.65	0.03	0.47	0.32	1.12	2.76	0.98	1.65	0.63	1.26	2.34
	Maximum deformations (x) in the orthodontic arch -[mm] in the lower teeth												
47	46	45	44	43	42	41	31	32	33	34	35	36	37
0.67	0.41	0.3	1.74	1.77	0.36	1.41	1.07	0.29	1.34	1.65	0.03	0.47	0.32

**Table 2:** Elastic constant values.

Elastic Constant (k) in the Orthodontic Archwire [N/m]							
	**17**	**16**	**15**	**14**	**13**	**12**	**11**
**0.012″**	277	100.6	273	212.9	142.9	176.8	70.9
**0.014″**	513	186.5	505.8	394.5	264.9	327.6	131.3
**0.016″**	875.6	318.2	863	673	451.9	559	224.1
	**21**	**22**	**23**	**24**	**25**	**26**	**27**
**0.012″**	81.6	142.2	108.9	158.6	153.2	60.6	105
**0.014″**	151.2	263.4	201.9	293.8	283.9	112.3	194.6
**0.016″**	258	449.5	344.5	501.3	484.3	191.7	332
	**47**	**46**	**45**	**44**	**43**	**42**	**41**
**0.012″**	199.5	102	208.2	130.6	107.3	261.6	410.5
**0.014″**	369.7	189	385.9	242	198.9	484.8	760.5
**0.016″**	630.7	322.6	658.3	412.9	339.3	827	1297.5
	**31**	**32**	**33**	**34**	**35**	**36**	**37**
**0.012″**	510.4	299.5	146.2	212.5	239.5	94.5	204.2
**0.014″**	945.6	555	270.9	393.7	443.8	175.1	378.3
**0.016″**	1613.2	946.8	462.2	671.7	757.1	298.8	645.5

**Table 3:** Descriptive statistical analysis of the elastic constant.

	Elastic Constant (k) in Archwire		
	**0.012** **″**	**0.014** **″**	**0.016** **″**
**Mean**	185.43	343.54	586.06
**Median**	155.93	288.88	492.81
**Minimum**	60.66	112.38	191.72
**Maximum**	510.41	945.60	1613.15

**Table 4:** Magnitude of force for each tooth for archwire diameters of 0.012″, 0.014″ and 0.016″.

Force Magnitude for Each Tooth							
	**1.7**	**1.6**	**1.5**	**1.4**	**1.3**	**1.2**	**1.1**
**0.012″**	0.5	0.1	0.25	0.1	0.24	0.21	0.16
**0.014″**	0.92	0.18	0.47	0.19	0.45	0.39	0.3
**0.016″**	1.58	0.3	0.79	0.32	0.77	0.66	0.5
	**2.1**	**2.2**	**2.3**	**2.4**	**2.5**	**2.6**	**2.7**
**0.012″**	0.09	0.39	0.11	0.26	0.1	0.08	0.25
**0.014″**	0.17	0.73	0.2	0.48	0.18	0.14	0.46
**0.016″**	0.29	1.24	0.34	0.83	0.31	0.24	0.78
	**3.1**	**3.2**	**3.3**	**3.4**	**3.5**	**3.6**	**3.7**
**0.012″**	0.55	0.09	0.2	0.35	0.01	0.04	0.07
**0.014″**	1.01	0.16	0.36	0.65	0.01	0.08	0.12
**0.016″**	1.73	0.27	0.62	1.11	0.02	0.14	0.21
	**4.7**	**4.6**	**4.5**	**4.4**	**4.3**	**4.2**	**4.1**
**0.012″**	0.13	0.04	0.06	0.23	0.19	0.09	0.58
**0.014″**	0.25	0.08	0.12	0.42	0.35	0.17	1.07
**0.016″**	0.42	0.13	0.2	0.72	0.6	0.3	1.83

**Table 5:** Force magnitude for archwire diameters of 0.012″, 0.014″ and 0.016″.

	Archwire Force 0.012″	Archwire Force 0.014″	Archwire Force 0.016″
**Mean**	0.19	0.36	0.62
**Median**	0.15	0.27	0.46
**Minimum**	0.007	0.01	0.02
**Maximum**	0.58	1.07	1.83

**Table 6:** Difference between the forces generated by archwires with different diameters of 0.014″–0.012″, 0.016″–0.014″ and 0.016″–0.012″.

Archwire Diameter	Force Magnitude			
	Mean	Median	Maximum	Minimum
**0.014** **″** **–** **0.012** **″**	0.17	0.13	0.49	0.01
**0.016** **″** **–** **0.014** **″**	0.25	0.19	0.76	0.01
**0.016** **″** **–** **0.012** **″**	0.42	0.32	1.25	0.02

**Table 7:** The physical and mechanical properties of the materials used in the simulation.

Component	Material	Density [Kg/m^3^]	Young’s Modulus [Pa]	Poisson’s Coefficient
Teeth	Enamel	2.958	7.79 × 10^10^	0.3
Periodontal ligament	Ligament	1.100	6.2 × 10^8^	0.42
Maxilla, mandible	Bone	1.400	1 × 10^10^	0.31
Bracket elements	Ni + Cr alloy	8.500	2.1 × 10^11^	0.31
Adhesive	Transbond XT	1.950	5 × 10^9^	0.3

**Table 8:** The values of the results obtained after the sensitivity analysis.

PDL Young’s Modulus [MPa]	Displacement [m]	Strain [m/m]	Stress [Pa]
620 MPa	3.2602 × 10^7^	0.00016369	10,939,000
62 MPa	3.7802 × 10^7^	0.00019041	10,924,000
6.2 MPa	4.501 × 10^7^	0.00022072	10,906,000

**Table 9:** The values of the results obtained after the mesh convergence analysis.

No. of Elements	Displacement [m]	Strain [m/m]	Stress [Pa]
250,905	1.0314 × 10^7^	5.1738 × 10^5^	3,454,900
325,342	1.1282 × 10^7^	5.0459 × 10^5^	3,734,300
886,244	1.1012 × 10^7^	5.4151 × 10^5^	3,639,100

**Table 10:** Distribution of mechanical parameters according to dental arch.

		Mean	Median	Minimum	Maximum
**Stress (MPa)**	**Mandible**	0.491	0.138	0.017	3.990
	**Maxilla**	0.214	0.120	0.013	1.345
**Displacements (μm)**	**Mandible**	0.0338	0.0085	0.0007	0.3113
	**Maxilla**	0.0359	0.0202	0.0049	0.122
**Deformations (με)**	**Mandible**	23.746	2.119	0.233	594.01
	**Maxilla**	2.664	1.542	0.064	19.392

**Table 11:** Distribution of mechanical parameters according to dental area.

		Mean	Median	Minimum	Maximum
**Stress (MPa)**	**Canine–canine**	0.666	0.421	0.018	3.990
	**Premolar–molar**	0.117	0.090	0.013	0.392
**Displacements (μm)**	**Canine–canine**	0.0502	0.0238	0.0051	0.3113
	**Premolar–molar**	0.0146	0.0119	0.0007	0.0465
**Deformations (με)**	**Canine–canine**	28.642	3.531	0.251	594.01
	**Premolar–molar**	1.627	1.251	0.064	5.687

**Table 12:** Distribution of mechanical parameters according to type of orthodontic arch.

		Mean	Median	Minimum	Maximum
**Stress (MPa)**	**0.012″**	0.119	0.046	0.013	0.519
	**0.014″**	0.300	0.140	0.017	1.199
	**0.016″**	0.638	0.283	0.071	3.990
**Displacements (μm)**	**0.012″**	0.0141	0.0082	0.0007	0.0985
	**0.014″**	0.0276	0.0155	0.0013	0.1825
	**0.016″**	0.0478	0.0265	0.0022	0.3113
**Deformations (με)**	**0.012″**	1.561	0.679	0.064	14.515
	**0.014″**	7.596	1.799	0.307	95.95
	**0.016″**	30.457	3.389	0.91	594.01

**Table 13:** The magnitude of the force as a function of tooth displacement at the level of teeth 32 and 13 for the archwires with a thickness of 0.012″, 0.014″, and 0.016″ and the difference between the forces applied to the two teeth.

Archwire	Tooth	Magnitude of Force as a Function of Tooth Displacement			
		0	0.5	1	1.5
**0.012″**	32	0.09	0.21	0.35	0.49
	13	0.24	0.27	0.32	0.38
**0.014″**	32	0.16	0.38	0.64	0.9
	13	0.45	0.5	0.59	0.7
**0.016″**	32	0.27	0.65	1.1	1.54
	13	0.77	0.85	1.01	1.19

**Table 14:** Optimal force for different types of tooth movement [72].

Type of Dental Movement	Optimal Force (N)
Tipping	0.35–0.60
Translation	0.70–1.20
Radicular movement/torque	0.50–1.50
Rotation	0.35–0.60
Extrusion	0.35–0.60
Intrusion	0.10–0.20

## Data Availability

The original contributions presented in this study are included in the article. Further inquiries can be directed to the corresponding authors.

## Abbreviations

The following abbreviations are used in this manuscript:

DMS	dento-maxillary system
FEM	finite element method
2D	two-dimensional
3D	three-dimensional
CAD	computer-aided design
CBCT	Cone Beam Computed Tomography
